# 2-Fluorofucose Attenuates Hydrogen Peroxide-Induced Oxidative Stress in HepG2 Cells via Nrf2/keap1 and NF-κB Signaling Pathways

**DOI:** 10.3390/life12030406

**Published:** 2022-03-11

**Authors:** Mengjue Tu, Xingshuo Fan, Jianan Shi, Shengnan Jing, Xiaole Xu, Yuqin Wang

**Affiliations:** 1 Department of Pharmacology, School of Pharmacy, Nantong University, Nantong 226001, China; dreamjojojo@sina.com (M.T.); Zhiduoxingxingxing@163.com (X.F.); sjn1234562021@163.com (J.S.); 1827011093@stmail.ntu.edu.cn (S.J.); 2 Key Laboratory of Inflammation and Molecular Drug Target of Jiangsu Province, Nantong 226001, China

**Keywords:** 2-fluorofucose, fucosylation, oxidative stress, Nrf2, NF-κB

## Abstract

Fucosylation is one of the most important glycan terminal modifications that affects multiple biological activities of proteins. 2-Fluorofucose (2FF), its specific inhibitor, has recently been reported to reveal numerous biological effects by blocking fucosylation both in vitro and in vivo. The current study aimed to evaluate the effect of 2FF on hydrogen peroxide (H_2_O_2_)-induced oxidative damage in vitro. In our study, treatment with H_2_O_2_ increased the level of fucosylation, and 2FF improved the cell viability in H_2_O_2_-treated HepG2 cells. Our study also showed that 2FF significantly decreased the overproduction of reactive oxygen species (ROS) induced by H_2_O_2_ and the activities of catalase, glutathione and Mn-superoxide dismutase were remarkably increased by 2FF pretreatment. Furthermore, 2FF attenuated H_2_O_2_-induced early mitochondria dysfunction. The second part of the study revealed that 2FF enhanced antioxidant capacity by affecting Nrf2/keap1 and NF-κB signaling pathways in HepG2 cells. Being pretreated with 2FF significantly increased the nuclear translocation of Nrf2 and simultaneously promoted the expression of downstream proteins, such as HO-1 and NQO1. Moreover, 2FF remarkably suppressed the expression of inflammation-associated proteins. Taken together, these data suggest that 2FF might have a potential therapeutic effect for oxidative stress.

## 1. Introduction

Oxidative stress is defined as the imbalance between reactive oxygen species (ROS) generation and clearance, which is involved in the development and progression of many diseases, including cancer, diabetes type II, and neurodegenerative and liver diseases [1,2,3]. The impairment caused by excessive ROS is generally thought to be the result of proteins, lipids and DNA damage, eventually leading to cellular dysfunction and cell death [2].

Many studies have shown that numerous defense systems are induced to relieve oxidative damage in vivo. The nuclear factor erythroid 2-related factor 2 (Nrf2) is considered to be one of the key factors in the cellular antioxidative defense system in vivo [2,4]. Normally, Nrf2 activity is depressed by binding to Kelch-like ECH-associated protein 1 (keap1) in the cytoplasm. Upon oxidative stress, Nrf2 breaks away from the Nrf2-keap1 complex and transports to the nucleus. Then, it encodes detoxifying enzymes and antioxidant enzymes, including NAD(P) H:quinine oxidoreductase 1(NQO1), heme oxygenase 1(HO-1) and glutathione-S-transferases (GST) [5]. 

The transcription factor nuclear factor-κB (NF-κB) is of central importance in a series of cellular processes, such as inflammation, cell differentiation, proliferation and apoptosis [6]. Recently, mounting evidence has demonstrated that there is a correlation between NF-κB and ROS. The activity of NF-κB is regulated by the level of cellular ROS, and the transcription of NF-κB-dependent genes in turn affects the level of intracellular ROS [5]. It has been reported that ROS activates the NF-κB pathway mainly via modifying phosphorylation of IκBα [7]. Simultaneously, the NF-κB pathway influences the level of ROS by upregulating antioxidant proteins, such as Mn-superoxide dismutase (Mn-SOD), glutathione peroxidase (GPx), and HO-1 [8].

In mammalian cells, most proteins are post-translationally modified by glycan structures, which affects the stability, folding and biological activities of proteins [9]. Fucosylation, which is catalyzed by fucosyltransferases (FUTs), is a common glycan terminal modification. On the cell surface, altered fucosylated structures are often associated with cancer, inflammation and autoimmunity [10]. For instance, compared with corresponding fucosylated mAbs, mAbs devoid of core fucose that catalyzed by FUT8 have a strong affinity with Fcγ receptor IIIA and higher antibody-dependent cellular cytotoxicity [11]. Because of the important biological roles of fucosylation, its specific inhibitors may have important applications in research and therapy [12,13]. 2-Fluorofucose, one such inhibitor, was recently reported to inhibit tumor cell adhesion, migration and proliferation by blocking fucosylation in vitro [14,15]. Moreover, administration with 2FF inhibited the activation of NF-κB and expression of vascular cell adhesion molecule-1 in the livers of sickle cell disease mice [16]. However, the role that 2FF exerts on oxidative stress remains unknown.

Hydrogen peroxide (H_2_O_2_), which plays important roles in cellular physiology, is a key ROS molecule in the process of multiple human diseases [17]. In mammals, H_2_O_2_-induced cell damage is considered to be the best characterization system of oxidative toxicity and is often used to screen antioxidative agents. Therefore, in the current study, we aimed to estimate the protective effect of 2FF against H_2_O_2_-induced toxicity in HepG2 cells and expound the possible mechanisms.

## 2. Materials and Methods

### 2.1. Reagents and Antibodies

2FF was provided by SynChem. Inc., Elk Grove Village, IL, USA. Hydrogen peroxide was purchased from Sangon Biotech (Shanghai, China). Catalase (CAT), glutathione (GSH) and malondialdehyde (MDA) test kits were provided by Nanjing Jiancheng Bioengineering Institute (Nanjing, China). The mitochondrial membrane potential (MMP) assay kit (JC-1 Kit), lactate dehydrogenase (LDH) kit, MTT cell proliferation and cytotoxicity kit and Mn-SOD activity kit were obtained from Beyotime Biotechnology, China. ROS Fluorescent Probe-Dihydroethidium (DHE) was purchased from Vigorousbio. CO., Ltd. (Beijing, China). Biotinylated aleuria aurantia lectin (AAL, B-1395, 1:3000) was purchased from Vectorlabs, Inc. (Burlingame, CA, USA). Antibodies against Nrf2 (ab62352, 1:1000), NQO1 (ab34173, 1:1000), keap1 (ab139729, 1:2000), NF-κB p65 (ab32536, 1:2000), NF-κB p65 (phospho S536, ab76302, 1:1000) and Nrf2 (phospho S40, ab76026, 1:2000) were from Abcam (Cambridge, UK). Antibodies against glyceraldehyde-3-phosphate dehydrogenase (GAPDH, 60004, 1:5000), HO-1 (10701, 1:2000), cyclooxygenase-2 (COX-2, 66351, 1:1000), IκBα (10268, 1:1000) and Histone (10856, 1:1000) were from Proteintech (Wuhan, China).

### 2.2. Cell Culture and Treatments

The HepG2 cells (ATCC, Manassas, VA, USA) were cultivated with 10% fetal calf serum plus DMEM medium (Gibco, Waltham, MA, USA), containing streptomycin (100 U/mL) and penicillin (100 U/mL), in a humidifier containing 5% CO_2_ at 37 °C. After incubating with or without 100 μM 2FF for 48 h, the cells were individually exposed to 600 μM H_2_O_2_ dilutions for 4 h. Then supernatant medium and cells were harvested for the next experiments. 

### 2.3. siRNA Transfection

The Nrf2 knockdown cell model was established by transfection with specific siRNA. In brief, 10^6^ HepG2 cells were plated in 6-well plates and transiently transfected with 70 nM of small interfering oligonucleotide (siRNA) against Nrf2 (Shanghai GenePharma Co., Ltd., Shanghai, China) using Lipofectamine 2000 (Thermo Fisher Scientific, Waltham, MA, USA) for 4 h. Thereafter, cells were allowed to recover in fresh media for 24 h according to the manufacturer’s protocol. The efficiency of Nrf2 knockdown was confirmed by Western blot assay.

### 2.4. Cell Viability Assay 

An MTT (3-(4,5-dimethylthiazolyl-2)-2,5-diphenyltetrazolium bromide) assay was used for the determination of cell viability according to the manufacturer’s protocol. Briefly, 2 × 10^4^ HepG2 cells were plated and administered on a 96-well plate. MTT was prepared into a solution with a final concentration of 5 mg/mL and then added to the 96-well plate. After incubating for 4 h, a Formazan solution was added to the plate and mixed. Subsequently, the plate was put into an incubator until the purple crystals of formazan completely dissolved. The absorbance was measured at 570 nm with a microplate reader.

### 2.5. Fluorescence Probe-Dihydroethidium (DHE) Staining

ROS production was detected by DHE staining as described by Sun et al. [18]. Briefly, 10^5^ HepG2 cells were placed in a 24-well plate for processing and then incubated with 5 μM DHE at 37 °C for 30 min. The nuclei were stained with DAPI, and photos were taken with a laser confocal fluorescence microscope.

### 2.6. Lactate Dehydrogenase Assay

The cytotoxicity effect was detected by the LDH release assay kit. Briefly, the cells were plated in a 96-well plate and treated with or without H_2_O_2_, then the LDH release agent was added to the plate 1 h before the test. After centrifuging, the supernatant was collected and incubated with LDH working solution in the dark. Thirty minutes later, the absorbance at 490 nm was measured with a microplate reader.

### 2.7. Measurement of MDA Levels

The MDA content was determined with the thiobarbituric acid (TBA) method. Briefly, the cell suspension was mixed with the reagents in the kit at 95 °C for 40 min, and then the absorbance at 532 nm was measured. The MDA content was calculated according to the formula.

### 2.8. Enzyme Activity Assay

HepG2 cells were collected 4 h after H_2_O_2_ treatment. The activities of CAT and Mn-SOD were detected by the ammonium molybdenum acid method and the WST-8 method with the corresponding commercial assay kits, respectively. After H_2_O_2_ administration, cells were collected and homogenized for analysis at 4 °C. For the determination of CAT activity, the cell suspension was mixed with the reagents in the kit at 37 °C for 1 min, and then the absorbance at 405 nm was measured. For the detection of Mn-SOD activity, the cell suspension and reagent mixture were incubated at 37 °C for 30 min, then the absorbance at 450 nm was measured. Subsequently, the enzyme activities were calculated according to the formula. The total protein concentration was used as the standard for all the results in each sample.

### 2.9. Mitochondrial Membrane Potential (MMP) Assay

MMP was detected by JC-1 assay according to the manufacturer’s protocol. Briefly, 10^5^ HepG2 cells were placed in a 24-well plate for processing. After washing with PBS, the prepared cells were incubated with JC-1 (10 μg/mL) working solution for 20 min at 37 °C. Then, the supernatant was removed, and the cells were washed twice with staining buffer at the end. Changes in mitochondria were observed with a confocal fluorescence microscope. The J-aggregates produced red fluorescence, and the monomer emitted green fluorescence.

### 2.10. Immunofluorescence

After being fixed with 4% paraformaldehyde for 20 min, the cells were washed with PBS 3 times. The cells were then treated with PBS containing 0.3% Triton-X 100 and blocked with 4% BSA for 1 h. After incubation with Nrf2 antibody overnight at 4 °C, HepG2 cells were washed 3 times. Alexa Fluor 488-conjugated anti-rabbit immunoglobulin G (ab150081) was added, and cells were incubated in the dark for 2 h. The nucleus was stained with DAPI and finally washed with PBS 3 times. The images were taken with a confocal fluorescence microscope.

### 2.11. Western Blot and Lectin Blot Assay

Total protein was extracted with radio immunoprecipitation assay (RIPA) lysis solution with phenylmethylsulfonyl fluoride (PMSF) on ice. All extracted proteins were quantified by the BCA method and denatured at 95 °C after mixing with loading buffer. Western blot assay was performed according to a previous report [19]. For lectin blot assay, the membranes were blocked with 3% bovine serum albumin (BSA) in TBST followed by specific lectins. The bands were finally visualized by an ABC Kit (Vector Laboratories). Image-J analysis software was used to quantify band intensity and calculate relative protein content. 

### 2.12. Statistical Analysis

Data were presented as mean ± standard deviation. GraphPad Prism 7.0 software was utilized for data analysis. Comparisons between multiple groups were performed by one-way ANOVA analysis of Tukey’s multiple comparison test, and *p* < 0.05 was considered statistically significant.

## 3. Results

### 3.1. 2FF Improved Cell Viability in H_2_O_2_-Treated HepG2 Cells

**Figure 1:**
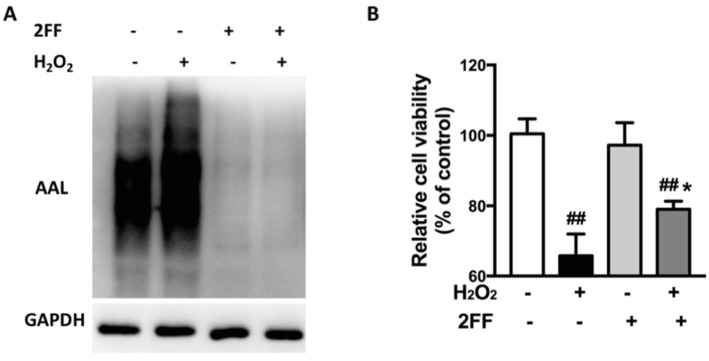
2FF improved cell viability in H_2_O_2_-treated HepG2 cells. After incubating with or without 100 μM 2FF for 48 h, the HepG2 cells were treated or not with 600 μM H_2_O_2_ for 4 h, then the cells were harvested. (**A**) The original figures of all western blot are provided in the Supplementary Materials. Lectin blot analysis using AAL was performed to recognize α1,6-fucose. GAPDH was used as a loading control. (**B**) The MTT assay was used to evaluate cell viability. Data were expressed as the mean ± SD of 3 separate experiments. ^##^ *p* < 0.01, vs. the control group without drug treatment. * *p* < 0.05, vs. the model group with H_2_O_2_ treatment.

Fucosylation is an important glycosylation modification that affects the function of numerous proteins [10,14]. Therefore, the lectin blot by probing with aleuria aurantia lectin (AAL), which preferentially recognizes α1,6-fucosylation, was used to test the effect of H_2_O_2_ treatment on fucosylation in HepG2 cells. The level of fucosylation was upregulated after H_2_O_2_ induction (Figure 1A), which suggested that altered fucosylation might affect the process of oxidative damage. The effect of 2FF on cell viability was detected by the MTT assay, and we found that 2FF remarkably increased cell viability after H_2_O_2_ treatment (Figure 1B). These results indicated that inhibition of fucosylation by 2FF might be beneficial for H_2_O_2_-injured cells. 

### 3.2. 2FF Alleviated Cell Damage and Loss of MMP in H_2_O_2_-Treated HepG2 Cells

**Figure 2:**
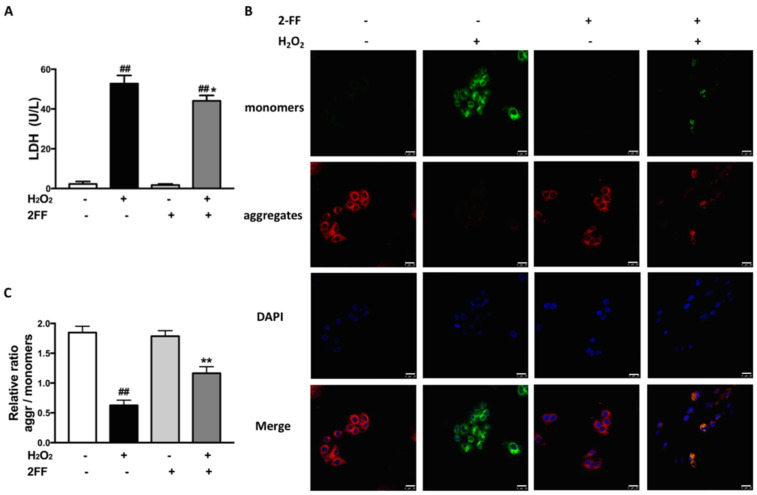
2FF alleviated cell damage and loss of mitochondrial membrane potential in H_2_O_2_-treated HepG2 cells. After incubating with or without 100 μM 2FF for 48 h, the HepG2 cells were treated or not with 600 μM H_2_O_2_ for 4 h, then the cells were harvested. (**A**) Cytotoxicity was evaluated in LDH assays for LDH leakage. (**B**,**C**) Mitochondrial dysfunction was indicated by the determination of mitochondrial membrane potential, which was measured by the JC-1 method and quantified using Image J analysis software. The monomer produced green fluorescence, and the J-aggregates emitted red fluorescence. DAPI was used for nuclear staining (blue). (Bar = 25 μm). Data were presented as the mean ± SD from 3 independent experiments. ^##^ *p* < 0.01, vs. the control group without drug treatment. * *p* < 0.05, ** *p* < 0.01, vs. the model group with H_2_O_2_ treatment.

Higher exposure of H_2_O_2_ leads to cell death by various mechanisms, then the intracellular enzyme release after the destruction of the cell membrane [17]. To investigate the effect of 2FF on H_2_O_2_-treated HepG2 cells, the content of lactate dehydrogenase (LDH) in the medium was measured to evaluate cell damage. Whether pretreated with 2FF or not, the levels of LDH significantly increased after H_2_O_2_ induction, while 2FF pretreatment significantly suppressed the increase induced by H_2_O_2_ (Figure 2A). Moreover, mitochondrial dysfunction is involved in the process of H_2_O_2_-induced oxidative damage [1,5]. As the marker event of early mitochondrial dysfunction, the loss of MMP was measured with JC-1 staining. After incubation with H_2_O_2_, the red fluorescence intensity of J-aggregates was significantly attenuated, suggesting mitochondrial dysfunction and damage. In contrast, pretreatment with 2FF effectively prevented H_2_O_2_-induced MMP loss (Figure 2B,C). These results further confirmed the protective effect of 2FF against oxidative damage in H_2_O_2_-induced HepG2 cells. 

### 3.3. 2FF Alleviated H_2_O_2_-Induced ROS Accumulation in HepG2 Cells

**Figure 3:**
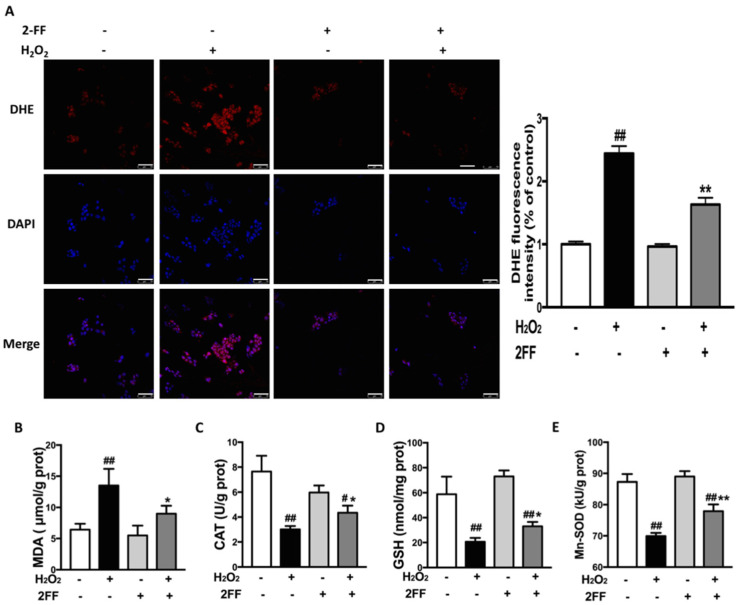
2FF alleviated H_2_O_2_-induced ROS accumulation in HepG2 cells. After incubating with or without 100 μM 2FF for 48 h, the HepG2 cells were treated or not with 600 μM H_2_O_2_ for 4 h, then the cells were harvested. (**A**) ROS in the cells was detected using a laser confocal microscope with DHE fluorescent dye (red) and quantified using Image J analysis software. DAPI was used for nuclear staining (blue) (Bar = 75 μm). (**B**–**E**) The levels of MDA (**B**), CAT (**C**), GSH (**D**) and Mn-SOD (**E**) were determined to evaluate the level of oxidative stress in HepG2 cells. Data were expressed as the mean ± SD of 3 separate experiments. ^#^ *p* < 0.05, ^##^ *p* < 0.01, vs. the control group without drug treatment. * *p* < 0.05, ** *p* < 0.01, vs. the model group with H_2_O_2_ treatment.

As a prominent factor related to liver diseases [3,20], excessive ROS also plays a fundamental role in the process of H_2_O_2_-induced cellular toxicity [4]. To explore the effect of 2FF on oxidative stress, the biomarkers of oxidative stress in HepG2 cells with different treatments were measured. Fluorescence probe-dihydroethidium (DHE) staining was used to determine the level of intracellular ROS. Meanwhile, the content of MDA was measured to reflect the level of lipid peroxidation in the cells. As expected, ROS generation and levels of MDA increased after H_2_O_2_ stimulation, whereas the effect of H_2_O_2_ was significantly suppressed by 2FF pretreatment (Figure 3A,B). Moreover, without H_2_O_2_ stimulation, 2FF pretreatment did not affect the generation of ROS in HepG2 cells. In addition, 2FF significantly ameliorated the reduction of GSH level, CAT and Mn-SOD activities in H_2_O_2_-treated cells (Figure 3C–E). These data indicated that 2FF reduced oxidative stress by suppressing the generation of ROS in H_2_O_2_-induced HepG2 cells. 

### 3.4. Inhibition of Fucosylation Influenced Nrf2/keap1 Signaling Pathway in H_2_O_2_-Treated HepG2 Cells

**Figure 4:**
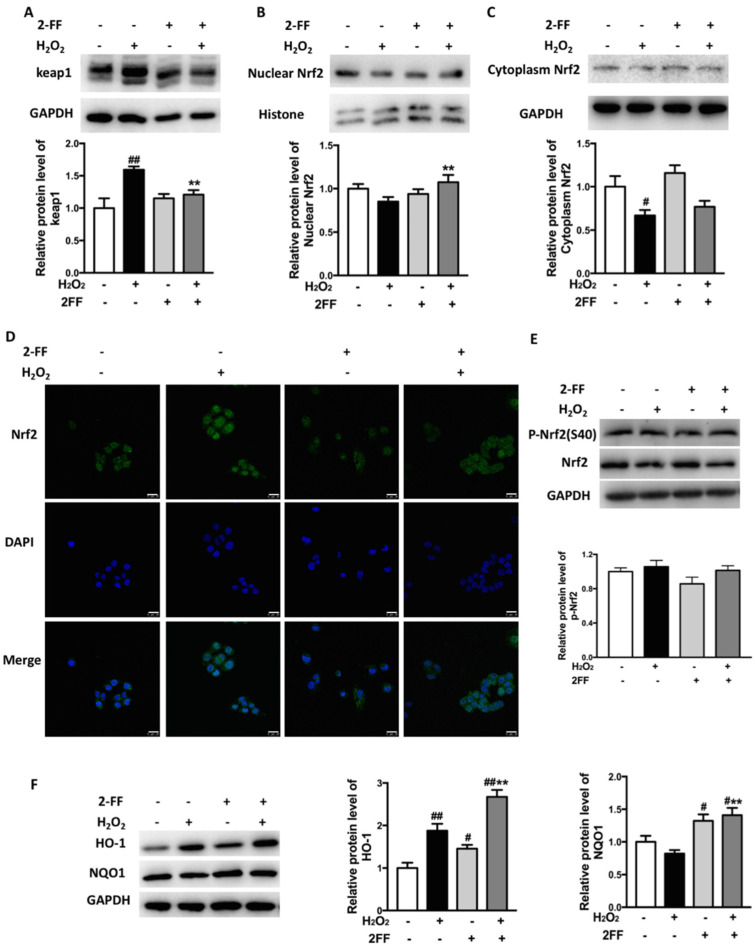
Inhibition of fucosylation influenced the Nrf2/keap1 signaling pathway in H_2_O_2_-treated HepG2 cells. After incubating with or without 100 μM 2FF for 48 h, the HepG2 cells were treated or not with 600 μM H_2_O_2_ for 4 h, then the cells were harvested. (**A**–**C**) Protein levels of keap1 (**A**), nuclear Nrf2 (**B**) and cytoplasm Nrf2 (**C**) were detected by Western blot. (**D**), Nrf2 translocation was indicated by immunofluorescent staining (green) and observed under a confocal microscope. DAPI was used for nuclear staining (blue) (Bar = 25 μm). (**E**,**F**), Protein levels of p-Nrf2 (**E**), HO-1, and NQO1 (**F**) were detected by Western blot analysis. The relative protein expression was quantified by densitometric analysis, with GAPDH, total Nrf2 or histone acting as controls. Data were expressed as the mean ± SD of 3 separate experiments. ^#^ *p* < 0.05, ^##^ *p* < 0.01, vs. the control group without drug treatment. ** *p* < 0.01, vs. the model group with H_2_O_2_ treatment.

Activation of the Nrf2/Keap1 system is clearly protective after H_2_O_2_ overexposure [5]. In order to explore the underlying antioxidant mechanism of 2FF, a Western blot assay was used to detect the protein levels of keap1, Nrf2, p-Nrf2, HO-1, and NQO1. Treatment of HepG2 cells with H_2_O_2_ significantly increased the expression of keap1, while 2FF treatment prior to H_2_O_2_ exposure attenuated the increase of keap1 (Figure 4A). The protein level of Nrf2 in the cytoplasm significantly decreased after H_2_O_2_ treatment, while that in the nucleus slightly decreased. However, pretreatment with 2FF upregulated the level of nuclear Nrf2, although it showed little effect on cytoplasmic Nrf2 (Figure 4B,C). This data was further confirmed by immunofluorescence (Figure 4D). Besides keap1, which is the major regulator of the protein stability of Nrf2, phosphorylation of Nrf2 is also involved in Nrf2 regulation. Unexpectedly, 2FF slightly decreased the level of p-Nrf2, and there was no significant difference compared with the control cells (Figure 4E). In addition, 2FF pretreatment significantly enhanced the protein levels of HO-1 and NQO1 compared to the H_2_O_2_ model group (Figure 4F). Thus, we suggested that the protective effect of 2FF on oxidative stress might be relate to the Nrf2/keap1 system, and Nrf2 was crucial for its cytoprotective mechanism. 

### 3.5. Knockdown of Nrf2 Eliminated the Protective Effect of 2FF against H_2_O_2_-Induced Oxidative Injury in HepG2 Cells

**Figure 5:**
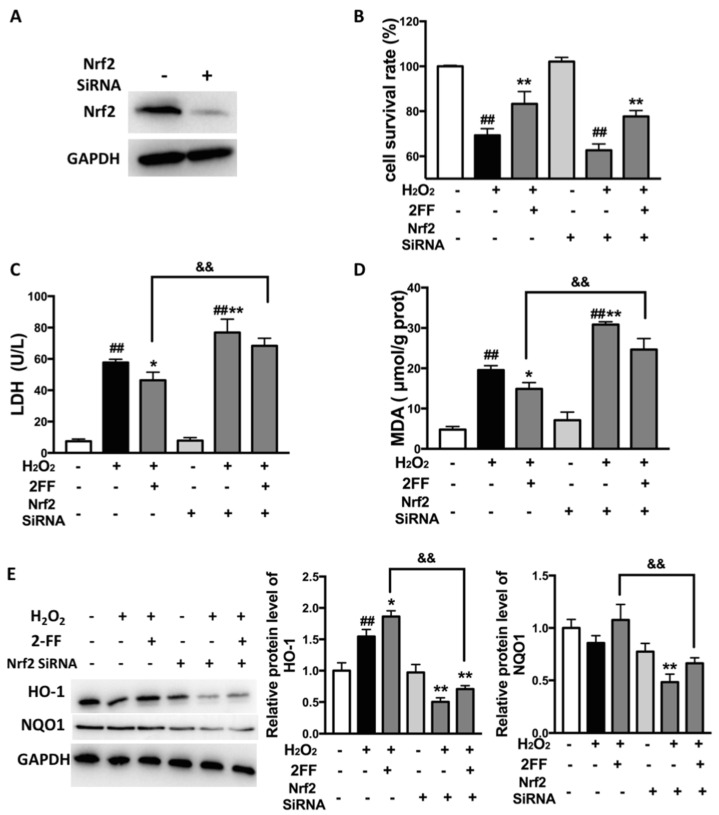
Knockdown of Nrf2 reversed the protective effect of 2FF on H_2_O_2_-injured HepG2 cells. The Nrf2 knockdown cell model was established by transfection with specific siRNA. (**A**) Western blot analysis was performed to confirm the efficiency of transfection. (**B**) The MTT assay was used to evaluate cell viability. (**C**) Cytotoxicity was evaluated by LDH assays for LDH leakage. (**D**) The level of MDA was determined for evaluating the level of oxidative stress in HepG2 cells. (**E**) Protein levels of HO-1 and NQO1 were detected by Western blot analysis. Quantification of relative protein expression was performed by densitometric analysis with GAPDH. Data were presented as the mean ± SD from 3 independent experiments. ^##^ *p* < 0.01, vs. the control group without drug treatment. * *p* < 0.05, ** *p* < 0.01 vs. the model group with H_2_O_2_ treatment. *^&&^ p* < 0.01 vs. 2FF-treated model group.

To verify our hypothesis on the mechanism of 2FF, the Nrf2 knockdown cell model was established by transfection with specific siRNA. Western blot analysis was performed to confirm the efficiency of transfection (Figure 5A). Cell viability was detected by the MTT assay, and we found that Nrf2 knockdown slightly reduced 2FF-increased cell viability in H_2_O_2_-treated cells (Figure 5B). Moreover, Nrf2 knockdown significantly enhanced the increase in LDH and MDA levels induced by H_2_O_2_ and partially abolished the reduction effect of 2FF on the levels of LDH and MDA in H_2_O_2_-treated HepG2 cells (Figure 5C,D). Subsequently, the protein levels of HO-1 and NQO1 were detected by Western blot analysis. As shown in Figure 5E, Nrf2 knockdown inhibited the protein expression of HO-1 and NQO1, especially after H_2_O_2_ treatment. However, 2FF pretreatment slightly alleviated the trend. All these results demonstrated that Nrf2 knockdown partially abolished the cytoprotective effect of 2FF, which also proved the important role of Nrf2 in the cytoprotective mechanism of 2FF.

### 3.6. Inhibition of Fucosylation Affected the Expression of Inflammation-Associated Proteins in H_2_O_2_-Treated HepG2 Cells

**Figure 6:**
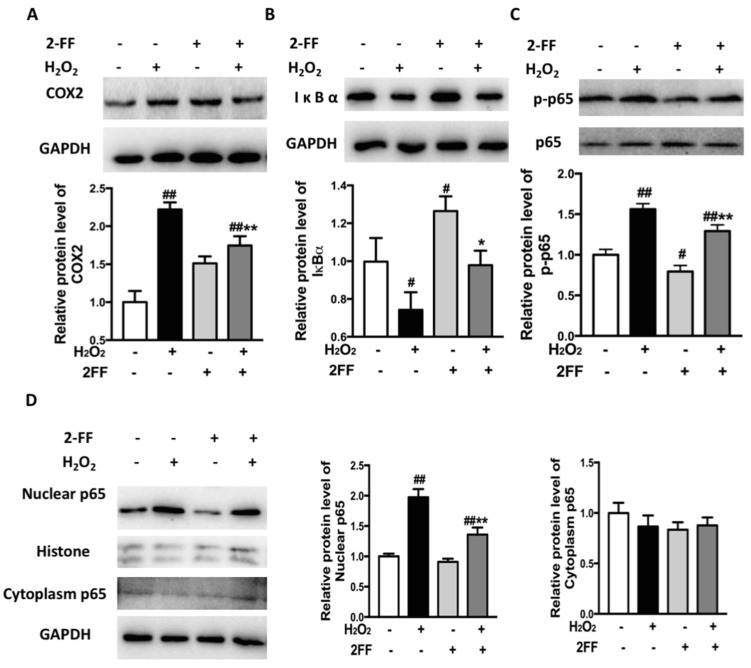
Inhibition of fucosylation affected the expression of inflammation-associated proteins in H_2_O_2_-treated HepG2 cells. After incubating with or without 100 μM 2FF for 48 h, the HepG2 cells were treated or not with 600 μM H_2_O_2_ for 4 h, then the cells were harvested. Western blot analysis was used to determine the expression of COX-2 (**A**), IκBα (**B**), p-p65 (**C**), nuclear p65, and cytoplasm p65 (**D**). The relative protein expression was quantified by densitometric analysis, with GAPDH, p65 or histone acting as controls. Data were expressed as the mean ± SD of 3 separate experiments. ^#^ *p* < 0.05, ^##^ *p* < 0.01, vs. the control group without drug treatment. * *p* < 0.05, ** *p* < 0.01, vs. the model group with H_2_O_2_ treatment.

Since inflammatory response plays an important role in oxidative processes [1], the protein expression of COX-2 was examined by the Western blot assay. The exposure of HepG2 cells to H_2_O_2_ produced a remarkable increase in COX-2, while pretreatment with 2FF induced a significant decrease in COX-2 levels (Figure 6A). According to previous studies, intracellular ROS regulates the NF-κB response, and NF-κB target genes affect the production of ROS [8]. To further explore the mechanisms of the cytoprotective effect of 2FF, the levels of IκBα and p65 were investigated. As illustrated in Figure 6B, the expression of IκBα was markedly decreased upon H_2_O_2_ treatment, meanwhile, 2FF attenuated the variation induced by H_2_O_2_. Concomitant with the inhibition of IκBα, the increased p65 induced by H_2_O_2_ was significantly suppressed in nuclei after treatment with 2FF in HepG2 cells (Figure 6D). In addition, whether there was H_2_O_2_ administration or not, the phosphorylation level of p65 was significantly reduced by 2FF pretreatment (Figure 6C). These results suggested that 2FF decreased H_2_O_2_-induced injury in HepG2 cells partially by inhibiting the activation of the NF-κB pathway. 

## 4. Discussion

In the current study, H_2_O_2_-injured HepG2 cells were used to evaluate the effect of 2FF on oxidative stress. Firstly, we found that H_2_O_2_ treatment upregulated the level of fucosylation in HepG2 cells and 2FF revealed a protective effect against H_2_O_2_-induced cell death. Then, the results demonstrated that 2FF ameliorated oxidative injury by enhancing the antioxidative system and inhibiting inflammatory-related proteins. 

The mitochondria play a crucial role in the process of ROS-mediated cell death [21,22]. As an early signal of mitochondria dysfunction, MMP significantly decreased in H_2_O_2_-treated cells, while 2FF ameliorated the situation (Figure 2). Simultaneously, the levels of ROS and MDA were consistent with these data. The burden of ROS production is largely counteracted by an intricate antioxidant defense system that includes the enzymatic scavengers SOD, CAT and GPx [5]. In the present study, the reduction of GSH level, CAT and Mn-SOD activities after H_2_O_2_ exposure was significantly alleviated by 2FF pretreatment (Figure 3). Accordingly, we speculated that inhibition of fucosylation by 2FF relieved oxidative damage in HepG2 cells. 

Activation of the Nrf2/keap1 system is clearly protective during oxidative stress [23]. Under oxidative stress, the dissociation of Nrf2 and keap1 increases with the accumulation of intracellular ROS, then the dissociated Nrf2 translocates to the nucleus and translates a set of antioxidant enzymes, such as NQO1, HO-1, and Mn-SOD [1,5]. Except for keap1, phosphorylation of Ser40 in the Neh2 domain of Nrf2 is also involved in Nrf2 regulation [5]. Several studies have suggested that oxidative stress will be alleviated when the expression levels of HO-1 and NQO1 are increased [24]. In the present study, although there was little effect on the cytoplasm Nrf2, 2FF increased the level of nuclear Nrf2. Unexpectedly, 2FF slightly decreased the level of p-Nrf2, and there was no significant difference compared with the control cells. As important antioxidant enzymes, expressions of HO-1 and NQO1 are generally considered to be regulated by Nrf2 [25,26]. However, Nrf2 is not the unique pattern used to regulate the expression of HO-1 and NQO1. For example, convincing evidence exists for HO-1 regulation by some other transcription factors, including HSF, AP-1 and NF-κB families [27]. In the present study, 2FF induced the increase of NQO1 and HO-1 expression in the absence and presence of H_2_O_2_ treatment (Figure 4F), which indicated that the protective effect of 2FF on oxidative stress might be partially related to Nrf2-independent induction of antioxidant enzymes. Moreover, Nrf2 knockdown partially abolished the reduction effect of 2FF on levels of LDH and MDA in H_2_O_2_-treated HepG2 cells, which also proved the important role of Nrf2 in the cytoprotective mechanism of 2FF (Figure 5). In short, these results provided evidence that the effect of 2FF on oxidative stress was partially related to the activation of Nrf2/keap1 system.

Recently, cumulative evidence has indicated that excessive ROS leads to the activation of the NF-κB pathway, while the NF-κB pathway also influences the expression of antioxidant proteins [8,24]. Therefore, we speculated that the NF-κB pathway might be involved in the antioxidant mechanism of 2FF. In response to specific stimuli, NF-κB first liberates itself from its inhibitory IκB partner, then translocates to nuclei and initiates the transcription of genes containing κB sites [6]. As a cellular DNA-binding subunit of NF-κB, p65 is probably the strongest activator of most genes with κB sites [6]. Our results showed that pretreatment with 2FF increased the level of IκBα and reduced the levels of p-p65 and nuclear p65 in HepG2 cells (Figure 6). These findings suggested that the protective effect of 2FF on oxidative stress was partly due to its inhibitory effect of NF-κB in HepG2 cells.

Since fucosylation is usually upregulated in various malignant tumors, previous studies on 2FF mainly focused on its effects on tumor growth and migration. For instance, Zhou et al. reported that 2FF suppressed HepG2 cell proliferation and migration, as well as tumor formation [15]. According to our findings, 2FF attenuated ROS generation and cell damage induced by high concentrations of H_2_O_2_. In this process, the Nrf2/keap1 system and the NF-κB pathway, as well as their downstream target proteins, likely contributed to the protective effect of 2FF. It has been well documented that ROS promotes the proliferation of cancer cells by activating survival pathways, including mitogenic signaling, but excessive ROS is harmful for cancer cells for the damage to cellular components [28]. Therefore, the overabundance of ROS and its induced cytotoxicity may be beneficial to cancer treatment [29]. From this perspective, 2FF may weaken the effects of some anticancer drugs that depend on ROS production, such as sorafenib [30].

Taken together, we elucidated the protective effect of 2FF against H_2_O_2_-induced oxidative stress and explored its possible mechanisms in the present study. Nevertheless, further studies are required to assess how 2FF regulates the activities of Nrf2 and NF-κB and to determine whether other signaling pathways can interfere with the antioxidant activity of 2FF.

## Data Availability

The data presented in this study are available in the main text.

## Supplementary Materials

The following are available online at https://www.mdpi.com/article/10.3390/life12030406/s1.
