# Supplementary figures and images for: 2-Fluorofucose Attenuates Hydrogen Peroxide-Induced Oxidative Stress in HepG2 Cells via Nrf2/keap1 and NF-κB Signaling Pathways
Source: Life (Basel). 2022 Mar 11;12(3):406. doi: 10.3390/life12030406 (PMC8950221; doi:10.3390/life12030406)

Figure S1

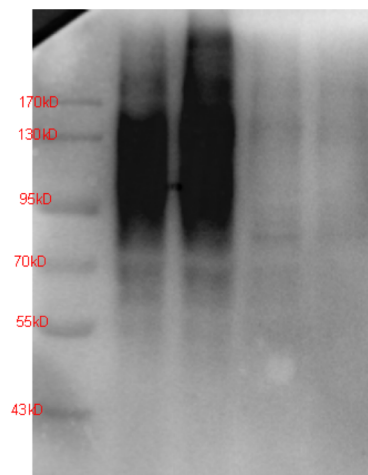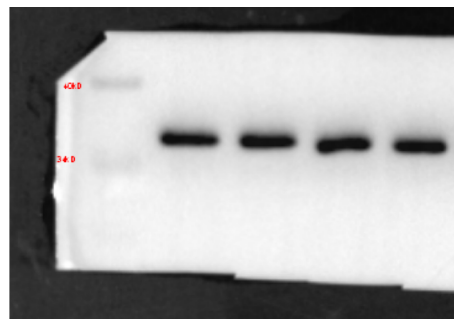

Figure S2

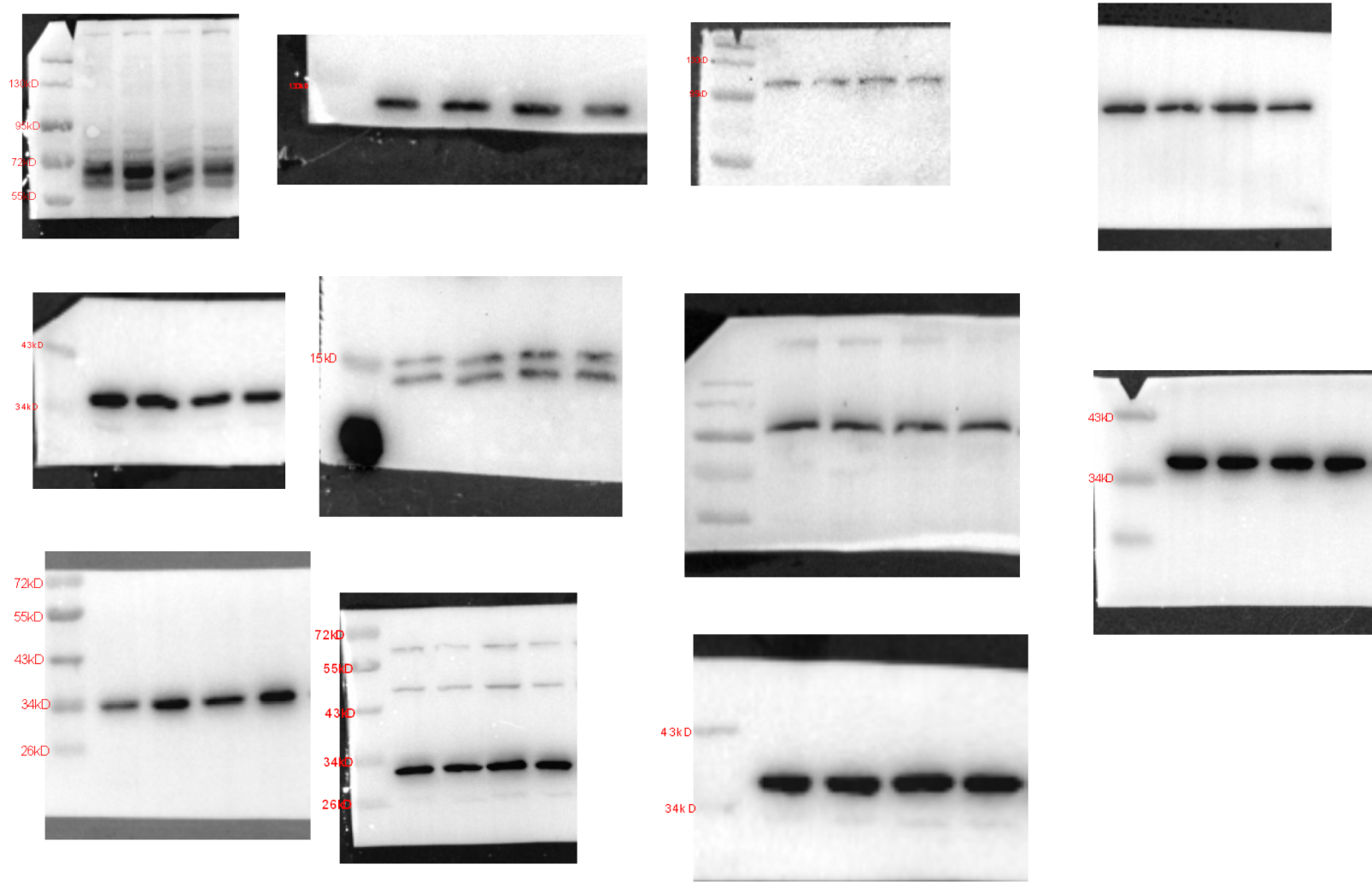

Figure S3

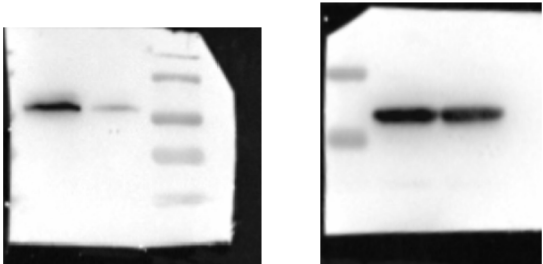

Figure S4

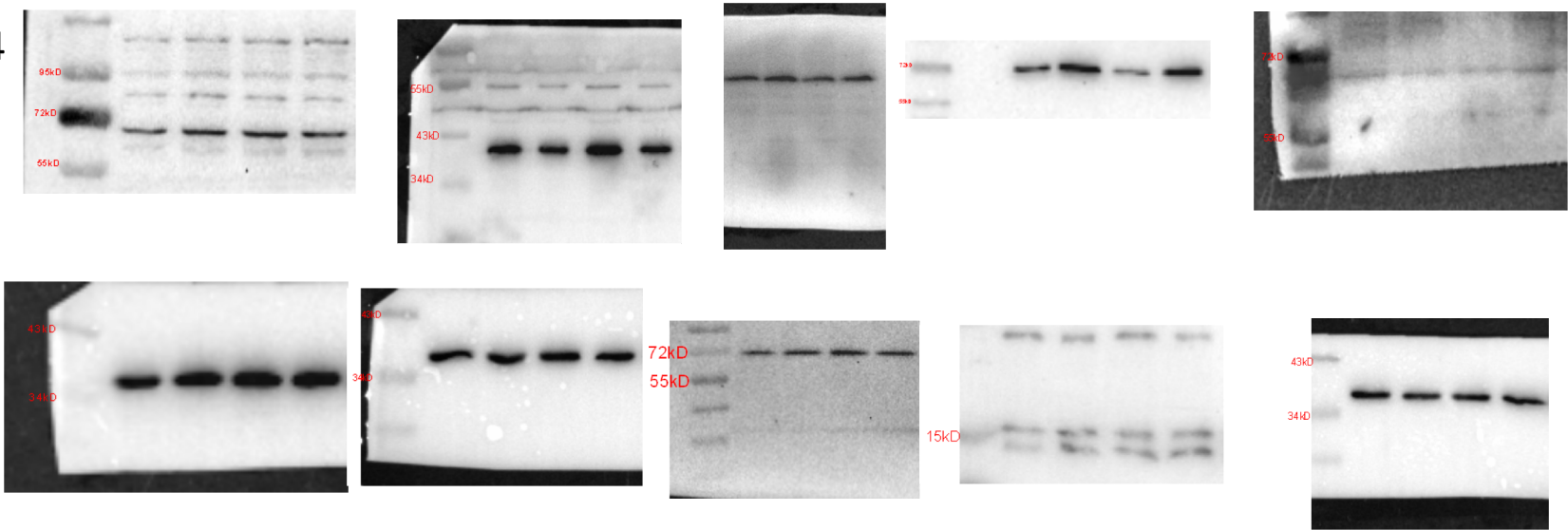

Supplement: Supplementary file 1 [file life-12-00406-s001.zip › life-1590842-supplementary.pdf]
